# BLASTPLOT: a PERL module to plot next generation sequencing NCBI-BLAST results

**DOI:** 10.1186/1751-0473-9-7

**Published:** 2014-03-31

**Authors:** Jesus Enrique Herrera-Galeano, Kenneth G Frey, Regina Z Cer, Alfred J Mateczun, Kimberly A Bishop-Lilly, Vishwesh P Mokashi

**Affiliations:** 1 Naval Medical Research Center-Frederick, 8400 Research Plaza, Frederick, MD, USA; 2 Henry M. Jackson Foundation for the Advancement of Military Medicine, 6720-A Rockledge Drive, Suite 100, Bethesda, MD 20817, USA

**Keywords:** BLAST, Plot, PERL, Package, Graph, Primer, Probe, NGS, Reads, Sequencing

## Abstract

**Background:**

The development of Next Generation Sequencing (NGS) during the last decade has created an unprecedented amount of sequencing data, as well as the ability to rapidly sequence specimens of interest. Read-based BLAST analysis of NGS data is a common procedure especially in the case of metagenomic samples. However, coverage is usually not enough to allow for *de novo* assembly. This type of read-based analysis often creates the question of how the reads that align to the same sequence are distributed. The same question applies to preparation of primers or probes for microarray experiments. Although there are several packages that allow the visualization of DNA segments in relation to a reference, in most cases they require the visualization of one reference at a time and the capture of screen shots for each segment. Such a procedure could be tedious and time consuming. The field is in need of a solution that automates the capture of coverage plots for all the segments of interest.

**Results:**

We have created BLASTPLOT, a PERL module to quickly plot the BLAST results from short sequences (primers, probes, reads) against reference targets.

**Conclusions:**

BLASTPLOT is a simple to use PERL module that allows the generation of PNG graphs for all the reference sequences associated with a BLAST result set.

## Background

A common task in genomics is the design of primers based on multiple sequence alignments (MSA) as well as the design of probes for microarray experiments. In both cases, either for PCR primers or for microarray probes, it is of crucial importance to ensure that the variance of the distance between the primers/probes is minimal. Often, the easiest way to ensure that the selected primers are evenly distributed is to inspect a plot of the reference sequence with the primers represented at their relative positions with regards to the reference sequence. A view of the particular area is important since it allows the identification of problematic areas or situations in which a single gap can easily be resolved by adding a primer/probe in a specific area. This procedure not only ensures that an even distribution of the primers is accomplished, but it also may save time by eliminating unnecessary runs of optimization software. In addition, in the case of NGS of metagenomic samples, the pathogen or agent of interest may be present in a proportion so small in comparison to the background (host or microbiome) that successful *de novo* assembly is unlikely [1]. The alternative is to run NCBI-BLASTN/X [2] using the filtered reads as queries against nt/nr NCBI–BLAST or a custom database [3]. In this case, it is important to visualize the distribution of the sequencing reads with relation to a particular reference [4,5].

Currently, there are several applications that allow for the visualization of BLAST results, such as BLASTVIEWER [6] and BLASTGRAPHIC [7]. By comparison, BLASTVIEWER requires an xml BLAST output which is obtained by specifying the appropriate option (−outfmt 5) when running BLAST. In addition, BLASTVIEWER only displays a particular set of high scoring segment pairs (HSPs) in relation to one reference (target) sequence at the time. Moreover, in our experience, BLASTVIEWER does not allow the observation of all the primers/reads matching a reference. Furthermore, even if seeing all the primers/reads related to a reference was possible, the user is obligated to take screenshots of each target sequence as the function to export all the plots appears to be currently unavailable. Alternatively, BLASTGRAPHIC [7] describes the desired functionality, but it is quite complex to install and run because it has many dependencies and several configuration steps. BLASTGRAPHIC is setup to run as part of a graphical front end for BLAST and not as an independent package. In order to run BLASTGRAPHIC, the user requires a local installation of BLAST, the Apache webserver and Bioperl among others. In addition, no documentation is available in the BLASTGRAPHIC website about how to run BLASTGRAPHIC independently from the graphical front end for BLAST. Furthermore, the URL that points to the BLASTGRAPHIC examples is no longer functional, indicating that support for the package has diminished.

Our recently developed tool, BLASTPLOT, displays all the primers/reads matching a reference sequence and it automatically outputs Portable Network Graphics (PNG) files for each reference sequence. It is simple to run with a driver ready to execute and its only dependencies are two standard PERL packages Math::Round and GD, both packages can be found in CPAN [8]. BLASTPLOT could be particularly helpful in the case of trying to generate primers from an MSA of closely related, but highly variable targets as described by Brodin et al. [9].

## Methods

BLASTPLOT was written in PERL, and takes advantage of two popular PERL packages, Math::Round and GD. BLASTPLOT is a PERL package with a subroutine called “new” that allows the user to run the module by simply creating a new instance of BLASTPLOT and calling the subroutine ‘plot’. The plot subroutine of BLASTPLOT requires only two parameters: the name of the BLASTN/X output file in table format and the name of the FASTA file that was used as a query. BLASTPLOT assumes that the “primers/reads” were used as the database. In other words, BLASTPLOT expects the first column on the blast output to contain the long sequences (reference) and it expects the second column to contain the short sequences (primers/reads). The program was written in the LINUX environment, but it can be run wherever the PERL interpreter is available with the Math and GD packages installed. An example of running BLASTPLOT after downloading the package could be “perl BLASTPLOT/nmrc_blastplot.pl output.blastn long_sequences.fa” where ‘nmrc_blast_plot.pl’ is a wrapper script calling BLASTPLOT, ‘output.blastn’ is the name of the BLASTN/X output file and ‘long_sequence.fa’ is the reference sequence FASTA file.

## Results and discussion

**Figure 1:**
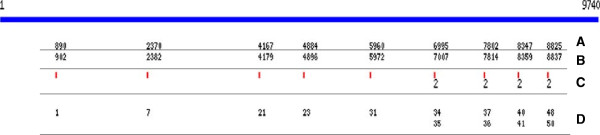
**Shows the distribution of primers for HIV-1 sequence.** A. CD.02._02CD_KTB035.AM000055, upper left corner, is the sequence identifier. 1, on the other middle left, is the start of sequence. 9740 on the middle right is the end of the sequence. **(A)** shows the position on the reference where the match with the query starts. **(B)** shows the position on the reference where the match with the query ends. **(C)** shows the count of primers that map to the same location (if it is greater the one). **(D)** shows the identifier for the query.

BLASTPLOT is a user-friendly PERL package which automatically generates PNG plot files for the locations of primers/reads in relation to each reference sequence used. Although this is a simple task, it can be tedious and, in our experience, there is no other package available that can accomplish this task without major investment of time by the user. BLASTPLOT does not need a complicated configuration and it can be easily run by both experienced and inexperienced users alike. Easily obtaining all the plots for the distribution of primers/reads in relation to reference sequences not only aids in the design of primers/probes and the analysis of NGS data, but it also facilitates the generation of plots that can be used for presentation and/or publication. As a concrete example, we used BLASTPLOT to generate plots for all the reference sequences during primer design for HIV-1. Figure 1 shows the primer distribution for one of the members of the MSA for HIV-1 [10]. In our experience, the plots were extremely useful in determining the areas where additional primers were required.

In addition, Figure 1 shows how the output from BLASTPLOT included the start and end coordinates for each match, the name given to the feature in the BLAST output, and the count of primers/reads mapping to the same coordinates.

## Conclusions

BLASTPLOT performs a very finite and simple task. However, it may reduce the time invested by users in performing similar tasks and it may be easily adopted into custom pipelines and modified as necessary. BLASTPLOT is freely available at https://sourceforge.net/projects/blastplot/.

### Availability and requirements

**Project name**: BLASTPLOT

**Operating systems(s)**: Linux and any other OS with PERL interpreter

**Dependencies**: PERL Math and GD packages

**Programming language**: PERL

**Any restrictions to use by non-academics**: No

### Availability

BLASTPLOT is available at https://sourceforge.net/projects/blastplot/.

## Abbreviations

NGS: Next generation sequencing; MSA: Multiple sequence alignments; PNG: Portable network graphics.

## Competing interests

The authors declare that they have no competing interests.

## Authors’ contributions

JEH identified the problem, designed the application, implemented the solution, and drafted the manuscript. KGF and KAB designed requirements for the applications. RZC tested the BLASTPLOT software and contributed to the writing. AJM and VPM contributed to the writing. All authors have read and approved the final manuscript.
